# Retrotransposition-competent L1s are increased in the genomes of individuals with amyotrophic lateral sclerosis

**DOI:** 10.3389/ebm.2025.10575

**Published:** 2025-06-03

**Authors:** Abigail L. Pfaff, Sulev Kõks

**Affiliations:** ^1^ Perron Institute for Neurological and Translational Science, Perth, WA, Australia; ^2^ Personalised Medicine Centre, Health Futures Institute, Murdoch University, Murdoch, WA, Australia

**Keywords:** retrotransposons, L1, amyotrophic lateral sclerosis, neurodegeneration, genetics

## Abstract

An individual’s genetics contributes to their risk of developing amyotrophic lateral sclerosis (ALS); however, there is still a large proportion of the heritability of ALS to be understood. Part of this missing heritability may lie in complex variants, such as the long interspersed element 1 (L1) retrotransposon, which have yet to be evaluated. The majority of L1 insertions in the human genome are no longer able to retrotranspose, but to date 279 retrotransposition-competent (RC) L1s have been reported. Many RC-L1s are polymorphic for their presence/absence; therefore, each individual will have a different number and complement of RC-L1s. These elements have been hypothesized to be involved in disease processes by multiple mechanisms such as somatic mutation by retrotransposition, the triggering of neuroinflammation and DNA damage. We hypothesize that L1s may influence disease development either through their effects on endogenous genes or through the properties that enable them to retrotranspose. Whole genome sequencing data from the New York Genome Center ALS consortium were used to characterize L1 variation identifying 2,803 polymorphic L1 elements and association analysis was performed in European individuals (ALS/ALS with other neurological disorder (ALSND) n = 2,653, controls n = 320). There were no individual L1 elements associated with disease, but we did identify a significant increase in the number of RC-L1s in ALS/ALSND genomes (p = 0.01) and the presence of ≥46 RC-L1s showed the most significant association (OR = 1.09 (1.02–1.16), p = 0.01) with disease. Analysis of individual L1s and their association with age at onset and survival identified one L1 whose presence was significantly associated with a lower age at onset (52.7 years) compared to homozygous absent individuals (59.2 years) (padj = 0.009). Our study has identified novel genetic factors for both disease risk and age at onset in ALS providing further evidence for the role of L1 retrotransposons in neurodegenerative diseases.

## Impact statement

This is the first study to assess genome-wide L1 variation in a large number of ALS and control genomes and to identify novel genetic factors involved in ALS risk and age at onset. ALS is a genetically heterogeneous disease with a large proportion of its heritability yet to be identified. Our study addressed this missing heritability by evaluating the presence/absence of the L1 retrotransposon and focusing on the subset of these elements that are still able to mobilize in the human genome. By understanding the mechanisms and risk factors that lead to ALS, new therapeutic targets can be identified for a disease with very limited treatment options.

## Introduction

**FIGURE 1:**
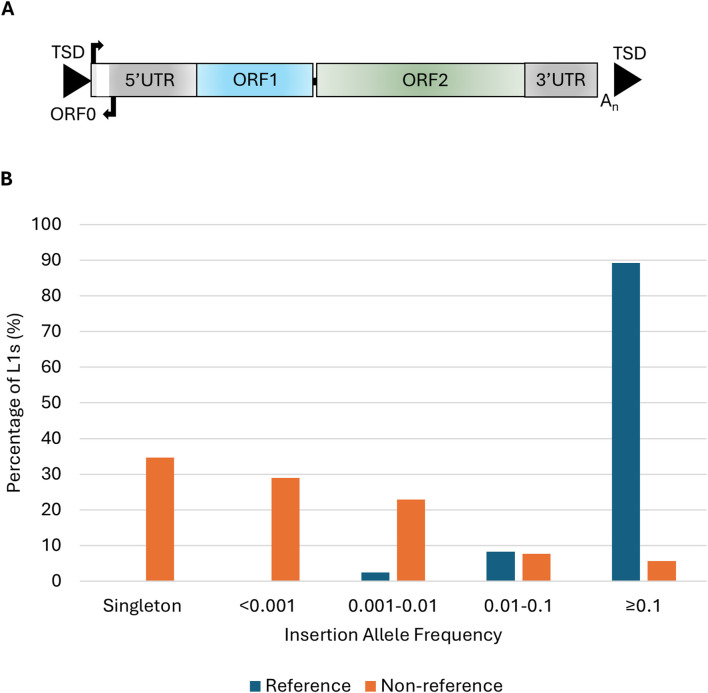
L1 structure and insertion allele frequencies of polymorphic L1s in the ALS consortium. **(A)** Schematic of a full-length L1 ∼6 kb in length that consists of a 5′untranslated region (5′UTR) containing the endogenous L1 promoter and an antisense promoter, three open reading frames (ORF0, ORF1, and ORF2), and a 3′untranslated region (3′UTR) flanked by target site duplications (TSDs). **(B)** The insertion allele frequency of 205 reference and 2,598 non-reference L1s in the NYGC ALS consortium (n = 4,393).

The long interspersed element-1 (L1) is the only autonomous family of retrotransposons in the human genome that contains elements currently able to mobilize and has played an important role in shaping the structure and function of the human genome [1]. L1s propagate through a “copy and paste” mechanism, termed target primed reverse transcription [2, 3], and mobilize the non-autonomous *Alu* and SINE-VNTR-Alu retrotransposons. The ongoing mobilization of L1s has led to genetic variation between individuals and can affect the function and expression of endogenous genes. There are at least 29 instances of disease caused by an L1 insertion, generally through loss-of-function mutations or aberrant splicing [4]. A full-length L1 element is ∼6 kb in size, contains both a 5′ and 3′-untranslated region (UTR), three open reading frames (ORF0, ORF1, ORF2), a poly A tail at its 3′end, and is flanked by variable target site duplications (Figure 1A). The proteins encoded by ORF1, a ∼40 kDa protein with RNA binding and chaperone activities, and ORF2, a ∼150 kDa protein with endonuclease and reverse transcriptase activities, are required for retrotransposition [5–8]. Despite L1s contributing to 17% of the human genome and more than one million L1s annotated in hg38 only 146 L1s are full-length with intact ORFs [9]. The majority are unable to mobilize due to internal deletions or rearrangements, 5′ truncations and mutations in the ORFs encoding the proteins required for retrotransposition [10, 11].

The dysregulation and elevated expression of L1s, along with other families of retrotransposons, have been detected in several neurological conditions including the neurodegenerative disease amyotrophic lateral sclerosis (ALS) [12, 13]. ALS, the most common form of motor neuron disease (MND), is a progressive disease that is characterized by the loss of motor neurons in the brain and spinal cord and the life expectancy after diagnosis is 2–5 years [14]. It is a clinically and genetically heterogeneous condition, and the neurodegenerative mechanisms involved are not fully understood. Several processes have been shown to be altered or dysregulated in the disease, such as protein aggregation, axonal transport, oxidative stress, mitochondrial dysfunction, RNA processing and the expression of retrotransposable elements [12, 15]. Changes in the expression of multiple classes and families of retrotransposons have been identified in subsets of individuals with ALS [16, 17]. An increase in expression of several families of retrotransposons was identified in the frontal cortex of individuals with ALS who carried the C9ORF72 expansion when compared to those without the expansion and healthy controls [16]. Another study using machine learning identified three subtypes of ALS based on transcriptomic data from the frontal and motor cortices, one of which was characterized by the activation of retrotransposons and TAR DNA-binding protein 43 (TDP-43) dysfunction [17]. Cytoplasmic accumulation of TDP-43, encoded by the gene TARDBP, in which mutations can cause ALS, is a hallmark of the majority of ALS cases. There is evidence in human tissues and cell lines that TDP-43 binds to retrotransposon transcripts of different classes, which is thought to aid in the repression of these elements [18]. Heterochromatin formation over repetitive elements, including L1s, acts to suppress their expression, but loss of nuclear TDP-43 from neurons results in chromatin decondensation over L1 elements [19].

We hypothesize that polymorphic L1s may play a role in ALS development either through their effects on endogenous genes, such as loss-of-function mutations, or through properties that are inherent in their ability to mobilize. These include somatic mutation through retrotransposition, the triggering of neuroinflammation via the interferon pathway and DNA damage through the endonuclease activity of the ORF2 protein [13, 20]. The ability of specific L1s to retrotranspose can be detected using cellular retrotransposition assays and by tracing the source elements of both germline and somatic insertions using 3′transductions [21–23]. Retrotransposition-competent L1s (RC-L1s) annotated in the human reference genome and non-reference RC-L1s can be polymorphic for their presence or absence. Therefore, each individual will have a different number and complement of RC-L1s in their genome, which could potentially lead to differing levels of functional L1 mRNA.

To investigate the role of L1s in ALS we utilized whole genome sequencing from the New York Genome Center (NYGC) ALS consortium to characterize the landscape of polymorphic L1s in ALS and control genomes to evaluate their effects on ALS risk, age at onset, and survival. These analyses were performed for both individual L1 insertions and the total number of RC-L1s present. We identified an increased burden of RC-L1s, those still able to mobilize, in the genomes of individuals with ALS and a single L1 associated with a reduction in the age at onset of the disease.

## Materials and methods

### Genotyping of L1s using whole genome sequencing from the ALS consortium

Whole genome sequencing data in cram file format aligned to hg38 were obtained from the NYGC ALS consortium. The ALS consortium includes individuals with a range of diagnoses such as ALS spectrum MND (ALS), other MND, other neurological disorders (including Parkinson’s disease and dementias) and ALS with other neurological disorders (ALSND) along with non-neurological controls (NNCs). The structural variant caller Delly^1^, with default settings, was used to identify structural variants in a subset of individuals (n = 244) [24]. The structural variants from each individual were merged and those deletions that overlapped with reference L1s were extracted. This generated a list of reference L1s that were absent in at least one of the 244 individuals. This panel of reference L1s was used in the second call step of Delly to generate genotypes for the entire ALS consortium cohort available (n = 4,393). Non-reference L1s were genotyped using Mobile Element Locator Tools (MELT version 2.2.2 in MELT-split mode) [22]. The L1 insertions detected were filtered to keep those supported by ≥2 split reads and an assessment score ≥3 and that had passed the filtering criteria performed by MELT. There were 210 reference and 2,649 non-reference polymorphic L1s identified in the ALS consortium cohort and after filtering for Hardy-Weinberg equilibrium (p <1 × 10^−6^ in NNCs) 205 reference and 2,598 non-reference L1s remained.

### Identification of retrotransposition competent L1s

RC-L1s were identified using published data on those L1s that displayed activity in a cellular retrotransposition assay or were identified as source elements for germline or somatic insertions using 3′ transduction events and details can be found in Pfaff et al [25]. The list of 198 RC-L1s from Pfaff et al has been extended using data from a more recent publication [26] and consists of 279 RC-L1s, 102 reference and 177 non-reference L1s.

### Association, age at onset and survival analysis

Association analysis was performed on those individuals who were >90% European according to the ALS consortium metadata and those diagnosed with ALS-spectrum MND or ALS-spectrum MND with another neurological condition (n = 2,653) and compared to non-neurological controls (n = 320) (see Table 1 for demographics). Association analysis of 501 polymorphic L1s (minor allele frequency >0.01) with ALS was performed using logistic regression adjusted for age, sex and sequencing preparation in PLINK (v1.07) and p values were adjusted for multiple testing (Bonferroni correction). Of the polymorphic L1s genotyped in the NYGC ALS consortium 106 were retrotransposition competent and 93 of these were polymorphic in the European subset. The 3 RC-L1s located on the X chromosome were removed from further analysis so that men and women could be compared and 1 RC-L1s was filtered out because >5% of genotypes were missing. Only individuals with genotypes for all 89 RC-L1s were retained for analysis. Linear regression adjusted for sex, age and sequencing preparation was used to analyze the association between the total number of RC-L1s present and disease status. In addition, NNCs and individuals with ALS/ALSND were categorized based on the number of alleles with an RC-L1 present to analyze the association between the likelihood of having ALS/ALSND and a certain number of RC-L1s. Logistic regression adjusted for sex, age and sequencing preparation was performed on these RC-L1 groupings to determine if having more than a certain number of these defined elements was associated with ALS/ALSND. P values were adjusted for multiple testing using Bonferroni correction.

**TABLE 1:** Demographics of the NYGC ALS consortium cohort in which association analysis was performed.

Demographic		NNC (n = 320)	ALS/ALSND (n = 2,653)
Gender	Male	157 (49.1%)	1,594 (60.1%)
	Female	163 (50.9%)	1,059 (39.9%)
Age^a^ Mean (min-max)		57.4 (17–90)	59.1 (12–90)

^a^ For NNC age at collection (43 unknown) and ALS/ALSND age at symptom onset (159 unknown).

Age at onset analysis was performed using linear regression of age at onset on L1 genotype or RC-L1 number with sex, sequencing platform and site of onset as covariates and p values were adjusted for multiple testing (Bonferroni correction). Survival analysis was completed using the Cox proportional hazards model from the ‘coxme’ package in R with sex, sequencing platform, age at onset and site of onset as covariates and p values were adjusted for multiple testing (Bonferroni correction). Individuals in the ALS consortium dataset who were still alive were censored at their last follow-up.

### L1s in ALS-associated loci

To identify polymorphic L1s from the ALS consortium located in ALS-associated loci the L1 coordinates were intersected with the coordinates of ALS-associated genes. The list of ALS associated genes was generated from the amyotrophic lateral sclerosis online database (ALSoD)^2^ using those defined as definitive ALS genes or with strong evidence for their association.

### Expression quantitative trait loci analysis in medial and lateral motor cortex

Matrix eQTL was used to calculate the L1 loci regulating the expression of transcript variants [27]. We used an additive linear model with covariates, age and sex, and FDR was used to correct multiple testing with only the results that remained significant after FDR correction (<0.05) reported here. Matrix eQTL also reports effect-size estimates as beta values or slope coefficients.

## Results

### The landscape of polymorphic L1s in the NYGC ALS consortium

In 4,393 whole genomes analyzed from the NYGC ALS consortium 205 reference and 2,598 non-reference L1s were identified as polymorphic for their presence/absence. The insertion allele frequencies (IAF) of the reference L1s were higher than those of the non-reference elements with 89.3% of the reference L1s having an IAF of ≥0.1 compared to 5.7% of the non-reference L1s (Figure 1B). The majority of L1 insertions were found in intergenic regions (59.5%) followed by 39.9% of the L1s found in introns. There were 3 (0.1%) L1 insertions located in exons, 2 (0.07%) in the 5′UTR and 12 (0.4%) in the 3′UTR of genes. The three exonic insertions were located in coding exons of the following genes *AASDH*, *HLA-DRB1* and *FSTL4*. The non-reference L1s located in the *AASDH* and *HLA-DRB1* genes were each found in a single individual diagnosed with ALS. The third exonic L1 is a common insertion (IAF = 0.77) found in the reference genome and the *FSTL4* transcript initiates from the antisense promoter of this L1 element. There were two L1 insertions located in the introns of ALS-associated genes *ERBB4* and *SCFD1* with IAF of 0.13 and 0.15 respectively in the ALS consortium, neither of which was associated with disease.

**FIGURE 2:**
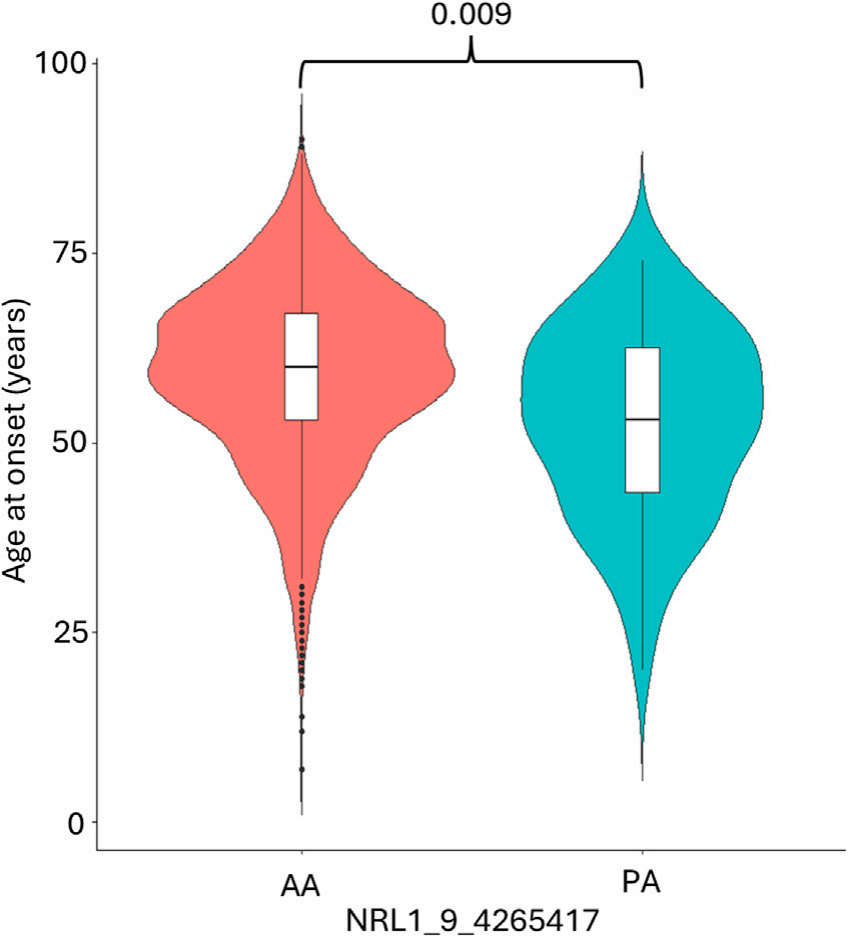
The presence of NRL1_9_4265417 is associated with a lower age at onset. The average age at onset of individuals diagnosed with ALS/ALSND was significantly lower in individuals with the PA genotype (52.7 years) compared to the AA genotype (59.2 years). PA n = 66 and AA n = 2,429.

**FIGURE 3:**
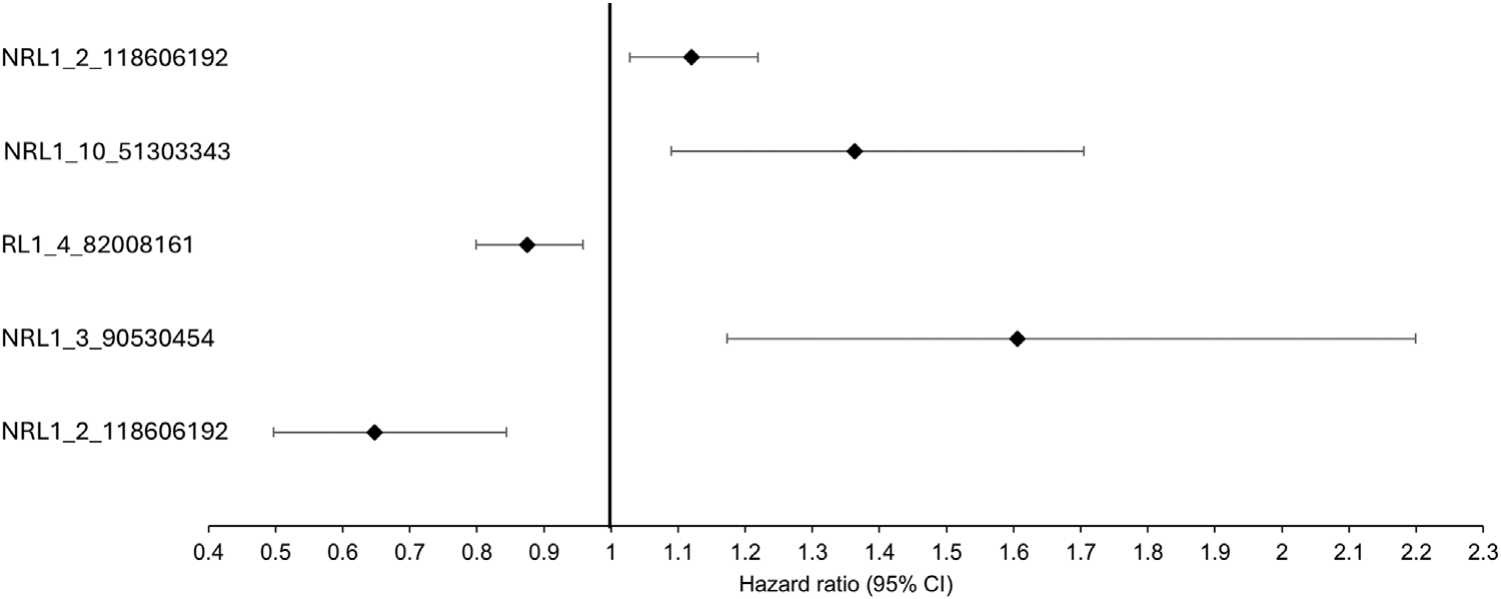
The top 5 most significant L1s associated with survival before correction. The black diamonds represent the hazard ratio and the black lines the 95% confidence intervals.

Association analysis was conducted on 501 L1 insertions (MAF >0.01) in the individuals of European descent in the ALS consortium as this formed the largest proportion of the cohort (Table 1 for demographics of this subset). There were 25 L1s associated with ALS/ALSND, but these did not survive correction for multiple testing (Supplementary Table S1). The top 5 L1s significantly associated with ALS/ALSND before correction are shown in Table 2. Age at onset analysis was performed identifying 31 associated L1s before correction with one L1 (NRL1_9_4265417) surviving correction for multiple testing (Supplementary Table S2). The presence of NRL1_9_4265417 was associated with a lower age at onset (est = -6.55, SE = 1.51, padj = 0.009). The average age at onset for individuals with the PA genotype was 52.7 years compared to 59.2 years for those with the AA genotype (Figure 2). NRL1_9_4265417 is a rare insertion with an IAF of 0.017 and located in intron 2 of the *GLIS3* gene. Survival analysis was performed identifying 26 associated L1s before correction for multiple testing (Supplementary Table S3), the top 5 of which are shown in Figure 3.

**TABLE 2:** Top 5 L1s significantly associated with ALS before correction.

L1 ID	Minor allele	MAF		OR (95% CI)	Unadj p value	Bonferroni p value
		NNCs	ALS/ALSND			
NRL1_20_43323466	P	0.431	0.371	0.76 (0.63–0.91)	0.003	1
NRL1_13_90973977	P	0.155	0.124	0.72 (0.57–0.92)	0.007	1
NRL1_20_29125080	P	0.025	0.014	0.46 (0.25–0.83)	0.011	1
NRL1_7_17055090	P	0.339	0.290	0.77 (0.64–0.94)	0.011	1
NRL1_10_89957889	P	0.081	0.105	1.54 (1.09–2.16)	0.013	1

### The number of RC-L1s is significantly increased in ALS/ALSND genomes

**FIGURE 4:**
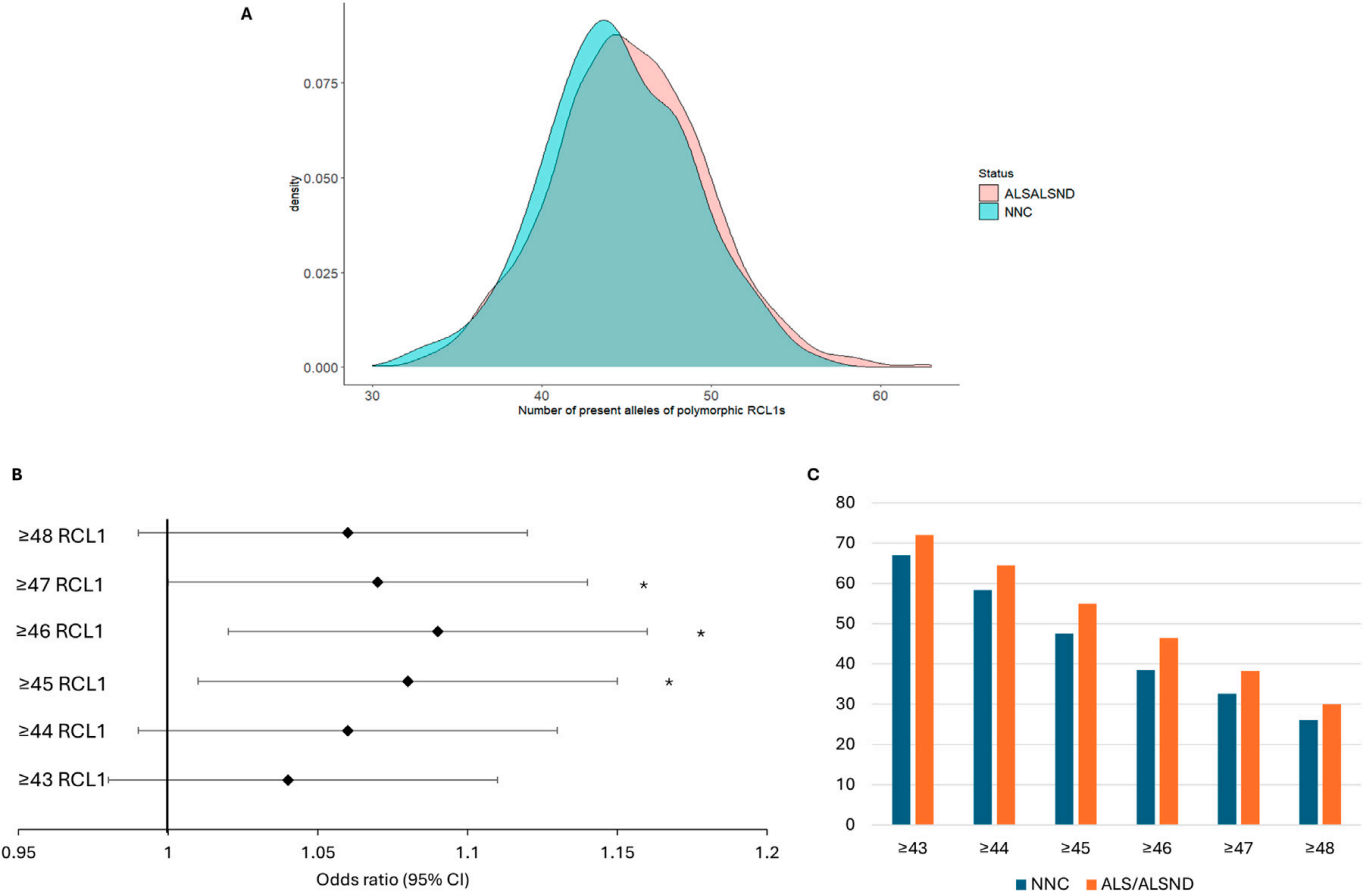
The total number of RC-L1s is increased in ALS/ALSND genomes. **(A)** The distribution of the total number of present alleles of 89 polymorphic RC-L1s in individuals diagnosed with ALS or ALSND compared to non-neurological controls (NNCs). **(B)** The forest plot represents the odds ratio of having ALS/ALSND based on an increasing number of polymorphic RC-L1s being present in an individual’s genome. The likelihood of having ALS/ALSND was significantly associated with having ≥45, ≥46 and ≥47 present alleles of the polymorphic RC-L1. The black diamonds represent the odds ratio, and black lines the 95% confidence intervals. **(C)** The percentage of individuals with a given number of RC-L1 alleles present. NNCs *n* = 288, ALS/ALSND *n* = 2,420. *p < 0.05.

In the NYGC ALS consortium 106 RC-L1s, 34 reference and 72 non-reference, were identified as polymorphic for the presence or absence from the compiled list of 279 RC-L1s. In the European subset of the cohort 93 RC-L1s were polymorphic. Not all the RC-L1s were detected as variable in our cohort as some were fixed in the population or were very rare and may only be present in specific population groups. The number of present alleles of 89 of the polymorphic RC-L1s in the European individuals, those on the sex chromosomes and with missing genotypes >5% were removed, was significantly higher in the genomes of individuals with ALS/ALSND compared to NNCs (β = 0.40, p = 0.01). The number of alleles present in NNCs genomes ranged from 32 to 56 and in ALS/ALSND genomes from 30 to 63 and their distribution is shown in Figure 4A. The ALS/ALSND and NNC individuals were grouped into 6 different categories to determine whether having more than a certain number of RC-L1s present at the 89 loci was associated with disease. The presence of ≥45, ≥46 and ≥47 polymorphic RC-L1s was significantly associated with ALS/ALSND (Figure 4B) and the percentage of individuals with a certain number of RC-L1s is shown in Figure 4C. The presence of ≥46 RC-L1s showed the most significant association (OR = 1.09 (1.02–1.16), p = 0.01) with 38.5% of NNCs having ≥46 compared to 46.5% of individuals with ALS/ALSND. The number of RC-L1s present in the genomes was not associated with age at onset or survival.

To determine whether the RC-L1s might influence ALS pathogenesis through effects on gene expression, an analysis was performed using genotypes of the 2,803 polymorphic L1s in this study and transcriptomic data from the medial and lateral motor cortex. This eQTL analysis identified 252 L1s that were significantly associated with expression changes of 395 different transcripts, but only 11 of these L1s were retrotransposition competent (Supplementary Table S4). The proportion of RC-L1s acting as eQTLs (4.4%) was not significantly different from the genome-wide proportion (3.8%) (Fisher’s exact test p = 0.60) suggesting that they are not enriched as eQTLs over those L1s that are not able to retrotranspose. There were 25 transcripts whose expression was associated with the 11 RC-L1s and the majority (72%) were either pseudogenes or novel transcripts with limited functional information available. Genes whose expression was associated with the presence/absence of RC-L1s included zinc finger protein 367 (ZNF367), olfactory receptor family 5 subfamily K member 1 (OR5K1) and maturin (MTURN).

## Discussion

Here we present data from a genome-wide analysis of polymorphic L1s using 4,393 whole genomes from the NYGC ALS consortium to identify novel genetic factors involved in ALS development and age at onset. ALS is a genetically heterogeneous disease with different patterns of inheritance and levels of gene penetrance with more than 30 genes associated with its development. The majority of large-scale studies have focused on single-nucleotide variants and suggest a heritability of 10–20%, which leaves 30–40% to be explained [28]. This missing heritability is likely to be found for other types of genetic variants and structural variants have already been associated with ALS [29]. Our study focused on polymorphic reference and non-reference L1s, evaluating their association with ALS as individual elements and in combination for those that can still mobilize within the human genome.

In the NYGC ALS consortium data 2,803 polymorphic L1s (205 reference and 2,598 non-reference) were identified. Association analysis performed on 501 L1s (MAF >0.01) did not identify individual L1s associated with disease after correction for multiple testing; however, when evaluating RC-L1s we identified an increased number of these elements in the genomes of individuals with ALS compared to controls. When analyzing the number of alleles present in 89 RC-L1s the total number of RC-L1s was higher in ALS/ALSND genomes (Figure 4A) and having ≥46 RC-L1s was most significantly associated with disease (Figures 4B,C). RC-L1s encode functional proteins that enable them to mobilize in the human genome and have the potential to generate somatic insertions, cause DNA damage and generate cytosolic RNA:DNA hybrids that activate the innate immune response and affect normal cellular function [13, 20]. These features of RC-L1s that are inherent in their ability to retrotranspose are important factors in how they may contribute to neurodegeneration and neuroinflammation. An increased number of these elements present in a genome could lead to higher levels of functional mRNA in a cell when these elements are expressed and potentially greater effects. Elevated expression of retrotransposons, including L1s, in ALS has been reported by multiple studies and was often restricted to a subset of individuals [12, 16, 17]. Analysis of locus specific expression of reference L1 elements encoding intact proteins showed an overall decrease in expression in the brains of individuals with ALS, but in a small number of individuals with ALS this expression was massively increased, up to 25 times the average [30]. This study was limited to reference L1s and included all L1s encoding intact proteins (not specifically those with evidence of their ability to retrotranspose); therefore, evaluation of both reference and non-reference RC-L1s would provide important data regarding their expression profile in ALS.

Methylation of a CpG island located in the L1 5′UTR is involved in regulating L1 expression and a reduction in methylation could indicate which elements are most likely to be expressed. For example, the expression of a specific L1 located on chromosome 13 occurred alongside its hypomethylation during hESC neurodifferentiation and in adult tissues such as the hippocampus [31]. Changes in methylation of L1s may also be associated with disease states, for example L1s were differentially methylated in the prefrontal cortices of individuals with psychiatric disorders [32]. When analyzing a small number of highly active RC-L1s we identified a reduction in the methylation of selected RC-L1s in the motor cortex of individuals with ALS compared to healthy controls [33]. A global evaluation of RC-L1 methylation and expression in ALS could help identify those elements that are altered in the disease to refine the list of RC-L1s used in our analysis. For example, when analyzing the burden of RC-L1s in Parkinson’s disease (PD) we identified an increased number of highly active RC-L1s in PD genomes rather than an increase in the presence of all RC-L1s [25].

Another potential mechanism by which RC-L1s may contribute to ALS pathogenesis is through their effects on endogenous gene expression. Transcriptomic analysis identified 252 L1s acting as eQTLs in either the medial or lateral motor cortex, of which 11 were RC-L1s affecting 25 different transcripts. The global association of RC-L1s as opposed to individual elements being linked to disease and the small number affecting gene expression suggest that it is their retrotransposition properties that are more likely to be involved in disease processes.

L1s, along with other retrotransposons, are therapeutic targets in a number of neurological conditions [34]. Reverse transcriptase inhibitors that target the L1 protein have been tested in a clinical trial for the neurodevelopmental disorder Aicardi-Goutieres syndrome [35, 36] and trials are currently underway in C9orf72 expansion positive individuals with ALS or frontotemporal dementia and in individuals with progressive supranuclear palsy. By analyzing RC-L1 variation and activity the aim would be to identify those individuals with ALS in whom L1s are contributing to disease development and who may benefit the most from these therapies targeting L1 activity.

We performed age at onset and survival analysis for both individual L1s and the burden of RC-L1s, identifying a single L1 (NRL1_9_4265417) whose presence was associated with a reduced age at onset (Figure 2). Age at onset of ALS is influenced by sex, family history and genetics [37]. Carriers of a Mendelian genetic variant have a lower age at onset and two recent age at onset GWAS have identified only two associated variants [28, 38, 39]. NRL1_9_4265417 is not in a known ALS-associated region and is located in intron 2 of the *GLIS3* gene, which is a transcriptional regulator and has been shown to modulate genes involved in autophagy and neuronal function [40]. Variants in *GLIS3* have also been associated with levels of tau and ptau in Alzheimer’s disease [41]. Analysis of the functional consequences of this L1 insertion will be required to determine its effects and how it may act as a disease modifier.

This study has identified novel genetic factors for both disease risk and age at onset in ALS providing further evidence for the role of L1 retrotransposons in this neurodegenerative disease and their potential as a therapeutic target.

## Data Availability

The WGS sequencing analysed in this study from the ALS consortium were obtained upon application to the New York Genome Center and data requests can be made by completing a genetic data request form at ALSData@nygenome.org. https://www.nygenome.org/science-technology/collaborative-research.

## Footnotes

1
https://github.com/dellytools/delly

2
https://alsod.ac.uk/
